# The moderating role of attachment styles in the relationship between psychological commitment and aggression among football fans

**DOI:** 10.3389/fpsyg.2026.1632498

**Published:** 2026-01-28

**Authors:** Serkan Volkan Sarı, Seher Pamaç, Sabır Sultanoğlu, Onur Göz

**Affiliations:** Department of Psychological Counseling and Guidance, Faculty of Education, Recep Tayyip Erdogan University, Rize, Türkiye

**Keywords:** aggression, attachment style, fans, football, psychological commitment

## Abstract

**Introduction:**

This study examined how attachment styles condition the relationship between football fans’ psychological commitment and aggressive tendencies.

**Methods:**

The sample consisted of 561 adult football supporters who identified themselves as long-term and highly committed fans. A moderation framework was employed to test whether attachment orientations shape the strength and expression of the association between psychological commitment and aggression.

**Results:**

Moderation analyses revealed that higher psychological commitment was generally associated with increased aggression; however, this relationship varied significantly as a function of attachment style. Secure attachment attenuated the positive association between commitment and aggression, whereas anxious ambivalent and avoidant attachment styles were associated with higher baseline levels of aggression.

**Discussion:**

Attachment styles function as regulatory lenses through which strong emotional investment in a team translates into either controlled or aggressive responses, offering important implications for prevention and intervention strategies in football environments.

## Introduction

In the contemporary globalized world, sports fandom has become an important element of individuals’ lifestyles, shaping how they express identity, loyalty, and emotional investment (Gordon et al., 2025). Although sports contribute positively to physical, mental, and social well-being (Butler, 2020; Guardian, 2024), intense identification with teams can also give rise to conflictual or aggressive behaviors. This issue is particularly salient in Türkiye, where football holds a culturally central position and fan-related incidents continue to attract national attention. For example, confrontations between rival fan groups, such as the post-match altercation following a Fenerbahçe-Galatasaray fixture or the historically significant “watery derby” between the same teams, illustrate how rivalry can escalate into collective aggression (NTV, 2024; Hürriyet, 2007; Berkowitz, 1987; Burton, 2004; Postmes et al., 2013; Wakefield and Sloan, 1995).

To interpret such reactions, it is essential to look beyond situational triggers and consider the individual-difference factors that shape how fans regulate emotions and respond to identity-related threats. In this regard, attachment theory offers a valuable perspective. Attachment styles are associated with differences in emotion regulation, threat perception, and interpersonal responsiveness factors closely tied to how supporters experience team-related successes, failures, and rivalries. Rather than uniformly producing similar outcomes, strong emotional investment in a team may lead to markedly different behavioral responses depending on fans’ attachment orientations. Despite their theoretical relevance, attachment styles remain underexplored in fan research, particularly within the Turkish context. Existing studies have largely examined commitment and aggression as direct outcomes of identification processes, paying less attention to how attachment-related regulatory tendencies may condition these associations. Therefore, the present study focuses on the conditional associations among fans’ psychological commitment, aggressive tendencies, and attachment orientations, with particular emphasis on the moderating role of attachment styles in shaping when and for whom psychological commitment translates into aggressive behavior. By adopting this perspective, the study aims to provide a more nuanced understanding of the psychological processes that contribute to both constructive and maladaptive supporter behaviors.

### Theoretical background linking fans’ psychological commitment and attachment styles

Sports fandom has long been recognized as a psychologically meaningful form of group affiliation. Although its historical roots can be traced back to ancient Olympic spectatorship (Lochbaum et al., 2024), contemporary perspectives conceptualize fandom primarily as a form of psychological commitment shaped by emotional, cognitive, and social investment in a team (Borland and MacDonald, 2003). Fan commitment is not a unitary construct; rather, it spans a continuum ranging from casual, entertainment-oriented involvement to deep and enduring loyalty that persists even during periods of poor team performance (De Groot and Robinson, 2008). Recent scholarship further highlights that fandom is increasingly embedded within identity-based and relational processes. As the commercialization of sport reshapes fan–team relationships into more symbolic and transactional forms (Giulianotti, 2002), group identification and identity-related psychological mechanisms have become central to explaining why supporters experience their teams as extensions of the self (Newson et al., 2026; Neville et al., 2022).

Psychological commitment reflects this multifaceted form of loyalty and is typically conceptualized through several interrelated dimensions, including personal identity, affective commitment, calculative commitment, social obligation, and regional or territorial identification (Matsuoka, 2001). Personal identity captures the extent to which individuals incorporate their team into their self-concept (Mael and Ashforth, 1992), whereas affective commitment reflects emotional attachment and enjoyment derived from team affiliation, paralleling affective bonds described in organizational commitment theory (Meyer and Allen, 1991). Calculative commitment refers to the perceived costs associated with disengaging from the team (Iverson and Buttigieg, 1999), while social obligation highlights normative expectations arising from fan communities (Lee and Park, 1996). Regional or territorial identification represents loyalty grounded in geographic and cultural belonging (Laverie and Arnett, 2000; Williams et al., 1992). Collectively, these dimensions underscore that psychological commitment is not merely behavioral but deeply relational and emotionally grounded.

Given this relational nature, attachment theory offers a useful framework for understanding individual differences in psychological commitment to sports teams. Attachment styles represent relatively stable orientations toward emotionally significant bonds and influence how individuals regulate closeness, dependency, and emotional investment in relational contexts. When applied to sports fandom, attachment orientations may shape how supporters emotionally engage with their teams and how consistently commitment is maintained as part of their identity. Secure attachment is generally associated with balanced emotional involvement, psychological stability, and coherent identity integration, which may facilitate stable and enduring forms of fan commitment. In contrast, insecure attachment orientations are characterized by less adaptive emotion-regulation strategies. Anxious-ambivalent attachment is often linked to heightened emotional sensitivity and dependency, potentially giving rise to fluctuating or tension-laden forms of commitment, whereas avoidant attachment is marked by emotional distancing and discomfort with closeness, which may be associated with weaker affective and normative investment in the team.

Although a broad range of demographic, social, and contextual factors including age, gender, cultural identification, team performance, and marketing practices have been shown to influence levels of fan engagement (Allison and Knoester, 2021; Clarke et al., 2024; Mastromartino et al., 2022; Yim et al., 2021), relatively little research has examined attachment styles as individual-difference variables associated with psychological commitment in sports fandom. Addressing this gap may enrich existing models of fan behavior by providing a more person-centered understanding of why supporters differ in the depth, stability, and emotional quality of their commitment. Importantly, examining the association between attachment styles and psychological commitment does not imply a causal or developmental sequence; rather, it allows for a theoretically coherent account of how stable relational orientations co-occur with distinct patterns of fan engagement. Consistent with this perspective, the present study proposes that psychological commitment is differentially associated with attachment styles.

*H*_1_: Psychological commitment is positively associated with secure attachment and negatively associated with avoidant and anxious-ambivalent attachment styles.

### Theoretical background linking attachment styles to aggression in sports fan behavior

Attachment theory provides a foundational framework for understanding how individuals regulate emotions and respond to relational threats. Originating from early caregiver–infant interactions (Bowlby, 1979, 2018), attachment styles give rise to internal working models that guide affect regulation, threat appraisal, and interpersonal behavior across the lifespan. Secure attachment is generally associated with emotional regulation capacity, psychological resilience, and adaptive social functioning (Shen et al., 2021; Verschueren, 2020; Goldberg et al., 1995; Meyer et al., 1993; Moretti et al., 2001; Fraley and Roisman, 2019). In contrast, insecure attachment orientations particularly anxious-ambivalent and avoidant styles are characterized by less effective regulatory strategies, heightened sensitivity to perceived threat, and greater vulnerability to maladaptive emotional responses (Bartholomew and Horowitz, 1991; Wanser et al., 2019). A substantial body of empirical research has linked insecure attachment to mood instability, chronic anger, low self-esteem, and heightened emotional reactivity, all of which are factors associated with increased aggression risk (Kawamoto, 2020; Lecompte et al., 2014; Mikulincer and Shaver, 2011; Passanisi et al., 2015; Levental O. et al., 2021). These attachment-related differences in emotion regulation are particularly salient in high-arousal social contexts, where threats to identity, status, or belonging may intensify aggressive tendencies.

Aggression within sports fandom particularly in football reflects complex interactions among psychological, social, and cultural processes. Traditional models distinguish between physical, verbal, and hostile forms of aggression (Berkowitz, 1993; Buss and Perry, 1992), whereas more recent perspectives emphasize identity-based mechanisms operating within group contexts. Strong identification with a team may intensify in-group favoritism and out-group derogation, thereby increasing the likelihood of aggressive responses during intergroup encounters (End et al., 2002; Wann et al., 2001). Research on hooliganism further demonstrates how identity fusion, rivalry salience, and collective emotional arousal create social conditions under which aggressive behavior becomes normalized or even valorized (Giulianotti, 1999). Within the Turkish football context, fan aggression has been shaped by regional affiliations, socio-political dynamics, and cultural norms related to masculinity and honor (Doğan, 2010; Koca and Bulgu, 2015; Yıldız, 2014), while situational factors such as crowd density and policing practices further influence behavioral outcomes (Çoban, 2014).

Integrating attachment theory with aggression research offers a theoretically coherent explanation for individual differences in aggressive tendencies among football fans. Anxious-ambivalent attachment is associated with hyperactivation of the attachment system, heightened threat vigilance, and emotional volatility, which may co-occur with reactive forms of aggression when fan identity is perceived as challenged. Avoidant attachment, characterized by emotional distancing and deactivating regulatory strategies, may be associated with more instrumental, displaced, or norm-violating expressions of aggression, particularly in deindividuated crowd settings where social accountability is reduced (Zimbardo, 1969). In contrast, secure attachment is linked to empathy, impulse control, and effective emotion regulation, which may constrain the escalation of emotional arousal into aggressive behavior. These attachment-based patterns are also consistent with social identity theory (Tajfel and Turner, 1986), which posits that emotionally charged group affiliations amplify responses to perceived intergroup threat, particularly among individuals with pre-existing regulatory vulnerabilities.

Despite extensive research on fan aggression, attachment styles remain underexplored as individual-difference variables within sports psychology. Prior studies have predominantly focused on demographic characteristics, personality traits, or sociocultural conditions, leaving a theoretical gap regarding how internal relational schemas are associated with aggressive behavior in fan communities. Examining attachment styles in relation to aggression provides an important foundation for understanding why some supporters are more prone to aggressive responses than others, independent of situational triggers. Addressing this gap contributes to a more psychologically grounded account of fan aggression and informs the development of prevention strategies that extend beyond crowd control and situational management.

*H*_2_: Aggression is negatively associated with secure attachment and positively associated with avoidant and anxious-ambivalent attachment styles.

### The moderating role of attachment styles in the relationship between psychological commitment and aggressive fan behavior

Research increasingly demonstrates that psychological commitment plays a central role in shaping how fans emotionally and behaviorally respond to competitive sport contexts. Higher levels of commitment have been associated with stronger anger reactions (Icekson et al., 2021), increased aggressive tendencies when belonging needs are heightened (Knapton et al., 2018), and elevated hostility under conditions of diminished self-confidence (Branscombe and Wann, 1992). Psychological commitment has also been shown to predict negative out-group emotions; for instance, Şirin and Şirin (2023) reported that commitment explains a substantial proportion of hostility toward rival teams. At the same time, engagement in team-related activities has been linked to enhanced psychological well-being and social connectedness (Cho et al., 2021; Yağız, 2020), while research on emotion regulation suggests that psychological commitment can simultaneously foster positive identification and heighten emotional sensitivity to competitive outcomes (Gross, 2015; Tamir M., 2022; Aytaç and Bilir, 2024). Indicating that psychological commitment can simultaneously foster positive identification and heighten emotional sensitivity to competitive outcomes. Identity-based processes further complicate this dynamic. Newson et al. (2026) demonstrated that club representation within national contexts strengthens identity fusion, intensifying in-group loyalty and shaping out-group attitudes in ways that may either constrain or escalate aggressive responses depending on contextual and individual factors.

Attachment theory provides a theoretically grounded explanation for why psychological commitment does not translate into aggressive behavior uniformly across individuals. Attachment orientations, shaped through early caregiver–child interactions (Bowlby, 1982; Mikulincer and Shaver, 2019; Santona et al., 2020), influence emotion regulation strategies, relational expectations, and coping patterns throughout adulthood (Besharat and Khajavi, 2013). In fan contexts, secure attachment is typically associated with emotional stability, reflective regulation, and adaptive coping, whereas insecure attachment orientations particularly anxious and avoidant patterns are characterized by heightened emotional reactivity, increased threat sensitivity, or emotional disengagement (Tamir I., 2022; Rees et al., 2023). These regulatory differences suggest that attachment styles are likely to shape how strongly psychological commitment is linked to aggressive behavior under conditions of heightened emotional investment (Tamir M., 2022).

From this perspective, attachment styles are best conceptualized as moderators that condition the strength and functional expression of the commitment–aggression association, rather than as intermediary mechanisms that transmit causal effects. Secure attachment may attenuate the likelihood that strong psychological commitment escalates into aggression by supporting emotional control, perspective-taking, and self-regulation. In contrast, insecure attachment orientations may heighten vulnerability to aggressive responses among highly committed fans by limiting effective regulation in the face of identity threat or competitive tension. Importantly, this moderation-based framework avoids assumptions about temporal precedence or developmental causality and is therefore more consistent with the cross-sectional design of the present study.

These attachment-based processes unfold within broader social and cultural systems that further shape fan behavior. Theoretical perspectives such as Social Identity Theory, the Elaborated Social Identity Model, and deindividuation theory emphasize how strong in-group identification, crowd dynamics, and perceived anonymity amplify emotional arousal and aggressive tendencies in group settings (Drury and Reicher, 1999; Tajfel and Turner, 1986; Zimbardo, 1969). Within such contexts, attachment styles function as individual-difference factors that influence how highly committed fans respond to identity threat and competitive pressure. Specifically, secure attachment may serve as a regulatory buffer, whereas insecure attachment styles condition the manner in which psychological commitment is expressed under emotionally charged circumstances.

*H*_3_: Attachment styles moderate the relationship between psychological commitment and aggression, such that secure attachment serves as a buffer, whereas insecure attachment styles condition the strength of this association.

## Method

### Research model

The research model of the present study examines the relationships among fans’ psychological commitment, aggression, and attachment styles within a correlational research design. Correlational designs are widely used in quantitative research to investigate the strength and direction of associations among variables without implying causal sequencing (Karasar, 2023). In this framework, psychological commitment is conceptualized as a key predictor of aggressive tendencies among football fans, while attachment styles are positioned as moderating variables that condition the strength and direction of this relationship.

**Figure 1:**
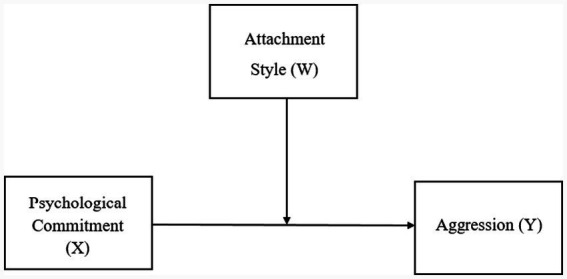
Moderation model illustrating the conditional effect of psychological commitment (X) on aggression (Y) as a function of attachment style (W).

Rather than assuming a causal or developmental sequence in which psychological commitment gives rise to attachment styles, the present model treats attachment orientations as relatively stable relational dispositions that shape how individuals regulate emotions and respond to identity-related threats. Accordingly, attachment styles are expected to influence when, for whom, and under what conditions psychological commitment translates into aggressive behavior. Specifically, secure attachment is expected to buffer the association between commitment and aggression, whereas insecure attachment styles particularly anxious-ambivalent and avoidant attachment are expected to amplify this association under conditions of heightened emotional investment and perceived group threat. Given that all variables were measured at a single time point, a moderation-based analytical framework was deemed conceptually and methodologically more appropriate than a mediation approach. This model allows for the examination of interaction effects between psychological commitment and attachment styles without imposing assumptions about temporal precedence or causal direction. The proposed research model is illustrated in Figure 1.

### Sample

The sample consisted of 561 adults who met a set of predefined inclusion criteria designed to identify individuals with long-term and stable fan identities. Demographic information, including age, gender, and team affiliation, is presented in Table 1. Participants ranged in age from 18 to 45 years (M = 22.48, SD = 3.57). For descriptive clarity, age was additionally grouped into four categories (18–25, 26–35, 36–45, and 45+), with the largest proportion of participants falling within the 18–25 and 26–35 age ranges. To qualify for participation, individuals were required to: (a) be 18 years of age or older; (b) self-identify, via an initial screening question, as loyal football fans for at least the past 10 years; (c) actively engage in fandom behaviors such as watching matches, following team-related news, or participating in in-person or online supporter interactions; (d) report no neurological or psychiatric condition that would impede completion of the assessment; and (e) voluntarily agree to participate. These criteria ensured that the sample comprised individuals whose fan identity was sufficiently established to align with the aims of the study. Because loyal football fans do not congregate within a single structured setting and no formal sampling frame exists for this population, a field-based convenience sampling strategy was employed. This approach is commonly recommended for accessing hard-to-reach populations lacking centralized registries or unified gathering points (Bonevski et al., 2014). Data were collected through face-to-face recruitment in natural supporter environments where football fans typically interact. Researchers visited supporter associations, areas surrounding stadiums prior to matches, cafés functioning as informal gathering spaces, and public social settings known for football-related discussions.

**Table 1:** Demographic characteristics of the sample.

Variable	Category	f	%
Sample size	Total (N)	561	100.00
Gender	Male	312	55.61
	Female	249	44.39
Team supported	Galatasaray	165	29.41
	Fenerbahçe	149	26.56
	Beşiktaş	84	14.97
	Çaykur Rizespor	27	4.81
	Others	136	24.24
Age groups	18–25	285	50.79
	26–35	170	30.29
	36–45	85	15.14
	45+	21	3.74
Age	Mean ± SD	22.48 ± 3.57	-
	Min–Max	18–45	-

This field-based recruitment strategy offered two methodological advantages. First, it enhanced ecological validity by engaging participants within their natural social context. Second, it allowed for on site verification of inclusion criteria, ensuring that only individuals who clearly met the definition of long-term loyal fans were included. Convenience sampling was selected for its practicality and efficiency in contexts where accessibility constraints and the absence of a sampling frame render probabilistic sampling methods infeasible (Etikan et al., 2016).

To ensure that the collected data were statistically sufficient for the planned analyses, both *a priori* and *post-hoc* power analyses were conducted. An a priori power analysis was performed using GPower 3.1.9.7 software prior to data collection to determine the minimum required sample size for the planned moderation analyses. The analysis was based on a multiple linear regression framework with an alpha level of 0.05, statistical power of 0.80, and a medium effect size (f^2^ = 0.15), indicating that a minimum of 55 participants would be sufficient. Following data collection, a *post-hoc* power analysis was conducted to further evaluate the adequacy of the sample size for moderation analyses involving different attachment styles. Psychological commitment was specified as the independent variable, aggression as the dependent variable, and secure, avoidant, and anxious-ambivalent attachment styles were separately modeled as moderators. The results indicated that a minimum of 73 participants was required for secure attachment (f^2^ = 0.11), 27 for avoidant attachment (f^2^ = 0.32), and 32 for anxious-ambivalent attachment (f^2^ = 0.27). With a total sample of 561 participants, the study substantially exceeded these minimum requirements, confirming the robustness and statistical adequacy of the sample for the intended moderation analyses.

### Procedure

The data collection process was conducted in three stages. In the first stage, permission to use the instruments intended for the study was obtained via email from their original developers or adaptors. In the second stage, ethical approval confirming the study’s adherence to ethical standards was obtained from the Ethics Committee for Social and Human Sciences. In the third and final stage, as explained above, the instruments were administered to the participants face-to-face by the researchers. Prior to the data collection process, participants were informed about the purpose of the study, the voluntary nature of their participation, and the procedures for completing the survey. Each participant was asked to complete a consent form. The data collection process took approximately 15 min per participant.

### Measurements

#### Demographic form

The demographic form included questions designed to identify participants’ basic characteristics and eligibility for the study. Specifically, participants were asked about their age, gender, and favorite football team. Additional screening questions assessed whether they were 18 years or older, had maintained loyal football fandom for at least 10 years, actively engaged in fandom-related behaviors (e.g., watching matches, following team news, or interacting with supporters), and reported no neurological or psychiatric conditions that could impede completion of the assessment.

#### The fans’ psychological commitment scale

The psychological commitment levels of fans were measured using the scale developed by Matsuoka (2001) and adapted to Turkish culture by Bozgeyikli et al. (2018). This 30-item scale utilizes a five-point Likert format (1 = strongly disagree to 5 = strongly agree) and comprises six sub-dimensions: personal identity, emotional commitment, resource cost, psychological cost, social obligation, and territorial commitment. The internal consistency reliability coefficients for the sub-dimensions were reported as 0.86 for personal identity, 0.84 for emotional commitment, 0.78 for resource cost, 0.89 for psychological cost, 0.81 for social obligation, 0.87 for territorial commitment, and 0.93 for the overall scale. Confirmatory factor analysis demonstrated that the items clustered coherently within the six-dimensional structure of the scale. Specifically, personal identity items (Items 1, 7, 13, 14, 19, 28) showed factor loadings ranging from 0.63 to 0.81, emotional commitment items (Items 2, 8, 20, 26, 29) loaded between 0.61 and 0.83, resource cost items (Items 3, 9, 16, 22) loaded between 0.48 and 0.82, psychological cost items (Items 4, 10, 15, 21) ranged from 0.60 to 0.82, social necessity items (Items 5, 11, 17, 23) loaded between 0.48 and 0.67, and regional commitment items (Items 6, 12, 18, 24, 27, 30) demonstrated the highest factor loadings, ranging from 0.84 to 0.91, confirming the robustness of the six-factor structure (Bozgeyikli et al., 2018). A sample item from the scale is: “Ending my relationship with the team would clearly be very stressful for me.” Higher scores on the scale indicate greater levels of psychological commitment.

#### The three-dimensional attachment styles scale

The scale, developed by Erzen (2016) assesses individuals’ attachment styles. It comprises 18 items distributed across three sub-dimensions (secure, avoidant, and anxious-ambivalent attachment). The scale employs a five-point Likert format, ranging from 1 (strongly disagree) to 5 (strongly agree). “Although the scale allows for the identification of a dominant attachment orientation based on the highest sub-dimension score, in the present study attachment styles were treated as continuous variables in line with the moderation-based analytical framework.” Reliability analysis yielded Cronbach’s alpha coefficients of 0.80 for avoidant, 0.79 for secure, and 0.71 for anxious-ambivalent attachment styles. Exploratory factor analysis demonstrated that the items clustered consistently within the three-dimensional structure of the scale. Specifically, avoidant attachment items (Items 1, 2, 3, 4, 5, 6) showed factor loadings ranging from 0.511 to 0.743, anxious-ambivalent items (Items 7, 8, 9, 10, 11, 12) loaded between 0.488 and 0.732, and secure attachment items (Items 13, 14, 15, 16, 17, 18) demonstrated loadings between 0.570 and 0.702, confirming the robustness of the scale’s factorial structure (Erzen, 2016). A sample item from the scale is: “I keep my distance from people because they might hurt me.” Higher scores on the scale indicate a greater tendency toward the respective attachment style.

#### The aggression inventory

The Aggression Inventory, originally developed by Olweus (1977) and later revised by Berman et al. (1993), was adapted into Turkish by Çelik and Otrar (2009). The inventory comprises 30 items, of which 20 are evaluated, and utilizes a five-point Likert scale ranging from 1 (not at all appropriate) to 5 (completely appropriate). It assesses four dimensions of aggression physical aggression, verbal aggression, impulsive aggression, and avoidance of aggression and demonstrates strong psychometric properties, with a Cronbach’s alpha coefficient of 0.823 and a test-retest reliability coefficient of 0.728. The scored items and their factor loadings from the Turkish adaptation study are reported as follows: physical aggression items loaded between 0.453 and 0.626 (Items 9, 11, 12, 13); verbal aggression items between 0.194 and 0.528 (Items 3, 4, 6, 7, 8, 16, 21); impulsive aggression items between 0.136 and 0.464 (Items 15, 18, 20, 24, 25, 28, 30); and avoidance of aggression items (Items 17 and 22) both at 0.409. A sample item from the scale is: “When someone tries to boss me around, I stand up to them firmly.” Item-level means were not provided in the Turkish validation study; however, the loading structure supports the four-factor model, and higher scores on the inventory indicate greater levels of aggression.

### Data analysis

Prior to data analysis, the data were assessed for adherence to parametric assumptions. Specifically, skewness and kurtosis values were examined to evaluate the normality of the distributions (Table 2). All variables demonstrated skewness and kurtosis values within conservative thresholds (±1.5), well below commonly accepted limits for normality (Kline, 2018), indicating no substantial deviations from normality. Pearson correlation analyses were conducted to examine the relationships among the study variables. To test the moderating role of attachment styles in the relationship between psychological commitment (independent variable) and aggression (dependent variable), simple moderation analyses were performed using PROCESS Macro version 4.1 (Model 1) for SPSS 25.0, based on Hayes’ bootstrap method. All confidence intervals were estimated using 5,000 bootstrap samples. Descriptive statistics, including minimum, maximum, mean, standard deviation, skewness, and kurtosis values, were computed using SPSS 25.0.

**Table 2:** The normality and reliability results for the scale levels.

Variable	Skewness	Kurtosis	Reliability
Physical aggression	0.51	−0.56	0.87
Verbal aggression	0.13	−0.47	0.87
Impulsive aggression	0.43	−0.17	0.85
Avoidance of aggression	0.16	−0.71	0.87
The aggression inventory	0.29	−0.22	0.88
Secure attachment	−0.78	1.18	0.79
Avoidant attachment	0.81	0.65	0.75
Anxious-ambivalent attachment	0.23	−0.35	0.74
Attachment styles scale	0.63	1.14	0.76
Personal identity	−0.07	−0.96	0.90
Emotional commitment	−0.34	−0.79	0.91
Resource cost	0.43	−0.70	0.92
Psychological cost	0.12	−1.17	0.90
Social obligation	0.35	−0.37	0.93
Territorial commitment	0.51	−0.93	0.96
Psychological commitment scale	0.09	−0.82	0.96

## Results

Table 3 presents descriptive statistics regarding the variables of the study, aggression, attachment and psychological commitment.

**Table 3:** Descriptive statistics of study variables (*N* = 561).

Variable	Mean	Min	Max	SD
Aggression inventory	54.62	20.00	96.00	14.16
Secure attachment	19.64	5.00	25.00	3.18
Avoidant attachment	16.15	7.00	35.00	5.74
Anxious-ambivalent attachment	16.72	6.00	30.00	5.03
Attachment styles total score*	52.51	27.00	90.00	9.15
Psychological commitment scale	86.56	31.00	150.00	29.11

*The total attachment score is reported for descriptive purposes only.

Table 3 presents the descriptive statistics for all study variables based on a sample of 561 participants. Mean scores indicate moderate levels of aggression and relatively balanced distributions across secure, avoidant, and anxious-ambivalent attachment tendencies. In addition to the attachment subdimensions, the total attachment score is reported for descriptive purposes only, providing an overall summary of attachment-related tendencies in the sample; however, all inferential analyses were conducted at the subdimension level in line with the study’s theoretical framework. The Psychological Commitment Scale showed substantial variability across participants, reflecting differing levels of identification and emotional investment in the team. Table 2 summarizes the psychometric properties of the measures. All subscales demonstrated acceptable reliability coefficients (*α* = 0.74–0.96), and skewness and kurtosis values fell within conservative thresholds (±1.5), well below the commonly accepted limits for normality (Kline, 2018). These results indicate no substantial deviations from normality and support the suitability of the data for subsequent moderation analyses.

According to Table 2, all scales demonstrated satisfactory psychometric properties. Reliability coefficients ranged from 0.74 to 0.96 across all subdimensions, indicating high internal consistency. Skewness and kurtosis values for the variables fell within acceptable limits (±1.5), suggesting no substantial deviations from normality. Overall, the scales used in the study showed adequate reliability and distributional suitability for subsequent analyses (Table 4).

**Table 4:** The moderating role of secure attachment in the relationship between psychological commitment and aggression.

Predictor	*B*	SE	*t*	*p*	LLCI	ULCI
Constant	24.00	5.20	4.62	< 0.001	13.80	34.20
Psychological commitment (X)	0.18	0.06	3.00	0.003	0.06	0.30
Secure attachment (W)	−1.10	0.40	−2.75	0.006	−1.89	−0.31
X × W	−0.006	0.003	−2.33	0.020	−0.011	−0.001

Model summary: *R* = 0.50, *R*^2^ = 0.25, F = 61.89, *p* < 0.001. *N* = 561. Dependent variable: Aggression.

A moderation analysis was conducted using PROCESS Model 1 to examine whether secure attachment moderated the association between psychological commitment and aggression among football fans. Results indicated that psychological commitment was positively associated with aggression (*B* = 0.18, *p* = 0.003), suggesting that higher levels of commitment were related to increased aggressive tendencies. Secure attachment also exhibited a significant main effect on aggression in a negative direction (*B* = −1.10, *p* = 0.006), indicating that higher attachment security was associated with lower aggression. Importantly, the interaction term between psychological commitment and secure attachment was statistically significant (*B* = −0.006, *p* = 0.020). This finding demonstrates that secure attachment moderates the relationship between psychological commitment and aggression. Specifically, as levels of secure attachment increase, the positive association between psychological commitment and aggression becomes weaker. In other words, secure attachment appears to function as a buffering factor, attenuating the extent to which heightened psychological commitment translates into aggressive behavior.

The overall model was statistically significant, explaining 25% of the variance in aggression (*R* = 0.50, *R^2^* = 0.25, *F* = 61.89, *p* < 0.001), indicating a moderate explanatory power (Table 5).

**Table 5:** The moderating role of avoidant attachment in the relationship between psychological commitment and aggression.

Predictor	*B*	SE	*t*	*p*	LLCI	ULCI
Constant	22.01	4.65	4.73	< 0.001	12.88	31.15
Psychological commitment (X)	0.20	0.05	3.95	< 0.001	0.10	0.30
Avoidant attachment (W)	1.37	0.28	4.92	< 0.001	0.83	1.91
X × W	−0.0065	0.0029	−2.24	0.025	−0.012	−0.001

Model summary: *R* = 0.50, *R*^2^ = 0.25, F = 62.95, *p* < 0.001. *N* = 561. Dependent variable: Aggression.

A moderation analysis using PROCESS Model 1 was conducted to examine whether avoidant attachment moderated the association between psychological commitment and aggression. Results indicated that psychological commitment was positively associated with aggression (*B* = 0.20, *p* < 0.001), suggesting that higher levels of commitment were related to increased aggressive tendencies. Avoidant attachment also showed a significant positive main effect on aggression (*B* = 1.37, *p* < 0.001), indicating that individuals with higher levels of avoidant attachment reported higher aggression overall. Importantly, the interaction between psychological commitment and avoidant attachment was statistically significant (*B* = −0.0065, *p* = 0.025), demonstrating that avoidant attachment moderates the relationship between commitment and aggression. Specifically, while aggression levels were generally higher among individuals with elevated avoidant attachment, the strength of the positive association between psychological commitment and aggression became less steep at higher levels of avoidant attachment. This pattern suggests that avoidant attachment conditions how psychological commitment relates to aggression, rather than serving a protective or buffering function. The overall model was statistically significant and accounted for 25% of the variance in aggression (*R* = 0.50, *R^2^* = 0.25, *F* = 62.95, *p* < 0.001; Table 6).

**Table 6:** The moderating role of anxious-ambivalent attachment in the relationship between psychological commitment and aggression.

Predictor	*B*	SE	*t*	*p*	LLCI	ULCI
Constant	24.00	5.20	4.62	< 0.001	13.80	34.20
Psychological commitment (X)	0.17	0.06	3.04	0.003	0.06	0.28
Anxious-ambivalent attachment (W)	1.20	0.31	3.87	0.001	0.59	1.81
X × W	−0.0070	0.0030	−2.33	0.020	−0.013	−0.001

Model summary: *R* = 0.47, *R*^2^ = 0.22, F = 52.34, *p* < 0.001. *N* = 561. Dependent variable: Aggression.

**Figure 2:**
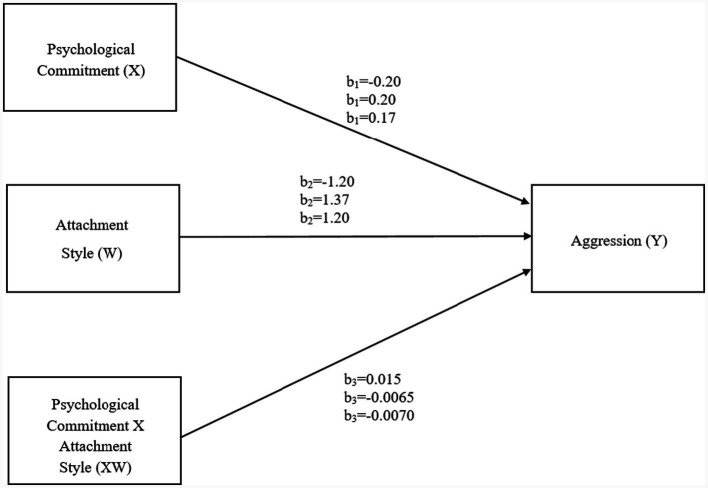
Moderation model illustrating the conditional effect of psychological commitment on aggression across attachment styles.

A moderation analysis was conducted using PROCESS Model 1 to examine whether anxious-ambivalent attachment moderated the relationship between psychological commitment and aggression. The results indicated that psychological commitment was positively associated with aggression (*B* = 0.17, *p* = 0.003), suggesting that higher levels of commitment were related to greater aggressive tendencies. In addition, anxious-ambivalent attachment showed a significant positive main effect on aggression (*B* = 1.20, *p* = 0.001), indicating that individuals with higher attachment anxiety reported elevated aggression overall. Crucially, the interaction between psychological commitment and anxious-ambivalent attachment was statistically significant (*B* = −0.0070, *p* = 0.020), demonstrating that anxious-ambivalent attachment moderates the association between commitment and aggression. Specifically, although aggression levels were generally higher among individuals with elevated anxious-ambivalent attachment, the positive relationship between psychological commitment and aggression became less steep as attachment anxiety increased. This pattern suggests that anxious-ambivalent attachment conditions how psychological commitment relates to aggression, rather than exerting a buffering or protective effect. The overall model was statistically significant and explained 22% of the variance in aggression (*R* = 0.47, *R^2^* = 0.22, *F* = 52.34, *p* < 0.001; Figure 2).

## Discussion and implications

In the present study, the first hypothesis addressed the theoretical association between fans’ psychological commitment and their attachment orientations. Although attachment styles were not modeled as direct predictors of psychological commitment in the empirical analyses, existing literature provides a robust framework for interpreting this relationship. Attachment theory posits that individuals’ relational orientations shape their capacity to form stable, emotionally meaningful bonds across a variety of social domains, including symbolic affiliations such as sports fandom. Accordingly, attachment styles may co-occur with distinct patterns of fan engagement without implying a causal or developmental sequence.

Prior research has highlighted structural and functional parallels between interpersonal attachment relationships and fan–team bonds. For example, Fink et al. (2022) identified conceptual similarities between attachment dynamics and fan identification processes, suggesting that insecure attachment orientations may be associated with less stable and more fragmented fan identities. In contrast, secure attachment has been linked to effective emotion regulation, social openness, and sustained group affiliation, characteristics that facilitate enduring psychological commitment to teams (Mikulincer and Shaver, 2019). Supporting this view, Levental A. et al. (2021) reported that secure attachment is associated with stronger fan commitment, while subsequent work demonstrated that collective identity processes play a central role in linking attachment security to fan loyalty (Levental A. et al., 2021). These findings align with broader evidence suggesting that identification with fan communities often mirrors relational dynamics observed in close interpersonal contexts (Cheche Hoover and Jackson, 2021; Grych and Kinsfogel, 2010).

Insecure attachment orientations may be associated with qualitatively different patterns of fan engagement. Avoidantly attached individuals tend to limit emotional closeness and may therefore approach fandom in a more instrumental or transactional manner, distancing themselves from intense affective involvement (Hare et al., 2009). Anxious-ambivalent attachment, by contrast, is characterized by heightened emotional dependency and sensitivity to rejection, which may result in fluctuating or tension-laden forms of commitment, particularly when expectations of belonging are threatened (Miga et al., 2010; Rom and Mikulincer, 2003). Developmental perspectives further suggest that early relational experiences and familial modeling shape the emotional scripts through which individuals later engage with collective identities, including sports fan communities (Enoch, 2003; Tamir I., 2022). Secure early attachments appear to facilitate social integration and stable group participation, whereas insecure attachment may constrain the formation of coherent and enduring fan identities (Santona et al., 2019).

Taken together, although the present study did not empirically test attachment styles as antecedents of psychological commitment, converging theoretical and empirical evidence supports the view that attachment orientations particularly avoidant and anxious-ambivalent patterns are meaningfully associated with variations in the depth, stability, and emotional quality of fan commitment. Securely attached fans, in contrast, appear more capable of sustaining consistent loyalty, engaging constructively with fan communities, and deriving psychological meaning from team affiliation. Framing attachment styles as co-occurring relational orientations rather than causal determinants is consistent with the cross-sectional design of the study and provides an appropriate conceptual foundation for interpreting the subsequent moderation findings.

Hypothesis 2 examined the association between attachment styles and aggressive tendencies among football fans. The findings of the present study indicate that attachment orientations are significantly associated with aggression, with anxious-ambivalent and avoidant attachment styles showing positive associations, whereas secure attachment is negatively associated with aggressive behavior. These results are consistent with a substantial body of empirical research demonstrating that insecure attachment is linked to heightened anger, hostility, and emotional reactivity across interpersonal and group-based contexts. Previous studies have shown that insecure attachment undermines the development of empathy and adaptive emotion regulation, both of which function as protective factors against aggression (Cricchio et al., 2022). Empirical evidence further suggests that individuals with anxious and avoidant attachment orientations exhibit elevated aggression and emotional dysregulation, largely due to difficulties in managing distress and perceived interpersonal threat (Karreman and Vingerhoets, 2012; Maalouf et al., 2022). In contrast, secure attachment has been consistently associated with lower levels of aggression, greater self-regulation capacity, and more constructive emotional expression (Kaplan, 2012; Kaplan and Aksel, 2013; Muarifah et al., 2022).

Attachment theory offers a coherent explanation for these patterns by emphasizing that anxiously and avoidantly attached individuals rely on less adaptive emotion-regulation strategies when confronted with stress. Anxious-ambivalent attachment is characterized by hyperactivation of the attachment system, heightened sensitivity to perceived rejection, and emotional volatility, which may increase susceptibility to reactive forms of aggression. Avoidant attachment, by contrast, involves deactivating strategies and emotional distancing, which may manifest in displaced, instrumental, or norm-violating expressions of aggression under conditions of interpersonal or identity threat (Simpson and Rholes, 2017). These regulatory patterns are particularly relevant in sports fandom, where emotionally charged group identification and rivalry contexts amplify threat perception and emotional arousal. Research has also shown that insecure attachment is associated with maladaptive relational scripts, including expectations of rejection and mistrust, which may predispose individuals to interpret ambiguous social cues as hostile and respond defensively (Rom and Mikulincer, 2003; Santona et al., 2019). Laverdière et al. (2013) further demonstrated that anxiously attached individuals are more likely to perceive interpersonal situations as threatening, thereby increasing the likelihood of aggressive responses. Neurobiological evidence supports these findings, indicating that insecure attachment is associated with heightened amygdala reactivity to social threat cues, potentially lowering the threshold for reactive aggression (Vrtička and Vuilleumier, 2012). Within the context of football fandom, these attachment-related vulnerabilities may translate into heightened aggression when team identity is challenged, rival groups are salient, or competitive outcomes threaten self-concept and belonging. Secure attachment, by contrast, appears to facilitate emotional regulation, empathy, and impulse control, thereby constraining the escalation of emotional arousal into aggressive behavior. Taken together, the present findings support Hypothesis 2 by demonstrating that attachment styles are meaningfully associated with aggressive tendencies, with secure attachment functioning as a protective relational orientation and insecure attachment styles associated with increased vulnerability to aggression.

Hypothesis 3 examined whether attachment styles moderate the relationship between psychological commitment and aggressive behavior among football fans. The findings provide clear support for a moderation-based interpretation, demonstrating that attachment orientations condition how psychological commitment translates into aggression rather than serving as intermediary mechanisms. Specifically, secure attachment buffered the positive association between psychological commitment and aggression, whereas anxious-ambivalent and avoidant attachment styles shaped the strength and functional expression of this relationship.

The buffering role of secure attachment suggests that securely attached fans are better equipped to regulate emotional arousal and manage identity-related threat, even when their psychological commitment to the team is high. Secure attachment has been consistently linked to adaptive emotion regulation, empathy, and impulse control, which may prevent strong identification from escalating into aggressive behavior (Hare et al., 2009; Mikulincer and Shaver, 2019). In the present findings, higher levels of secure attachment attenuated the slope of the commitment–aggression association, indicating that secure relational orientations constrain the extent to which heightened commitment is expressed through hostility or aggression. This pattern is consistent with theoretical models emphasizing the role of attachment security in promoting reflective coping and prosocial engagement under stress.

In contrast, anxious-ambivalent and avoidant attachment styles were associated with higher baseline levels of aggression, yet the positive association between psychological commitment and aggression became less steep at higher levels of these insecure orientations. This pattern suggests that insecure attachment does not amplify the commitment–aggression link in a linear manner but rather conditions how aggression is expressed among highly committed fans. For anxiously attached individuals, heightened emotional sensitivity and hyperactivation of the attachment system may produce elevated aggression regardless of commitment level, reflecting chronic regulatory vulnerability. Avoidantly attached individuals, who rely on deactivating strategies and emotional distancing, may exhibit aggression in more instrumental or displaced forms that are less directly contingent on fluctuations in commitment intensity. These findings highlight an important distinction between baseline aggression and the slope of the commitment–aggression relationship. Insecure attachment styles appear to elevate general susceptibility to aggression, whereas secure attachment operates to regulate how emotional investment is behaviorally expressed. Within highly arousing fan environments, such as intense rivalries or identity-threatening match outcomes, insecure attachment may render individuals more prone to aggression independently of incremental increases in psychological commitment. This interpretation aligns with social identity and intergroup emotion theories, which emphasize that perceived group threat and collective emotional arousal can elicit hostility even in the absence of additional motivational input (Branscombe and Wann, 1992; Mackie et al., 2000).

Conceptualizing attachment styles as moderators rather than mediators offers a theoretically coherent framework that avoids assumptions about causal sequencing and is consistent with the cross-sectional design of the study. Rather than transmitting the effects of commitment onto aggression, attachment orientations shape the conditions under which psychological commitment is more or less likely to manifest as aggressive behavior. This perspective also integrates well with prior research highlighting the roles of emotion regulation, identity threat, and group-based anger as parallel mechanisms contributing to fan aggression (Gross, 2015; Icekson et al., 2021; Levental A. et al., 2021; Taylor et al., 2007). Overall, the findings support Hypothesis 3 by demonstrating that attachment styles function as regulatory lenses through which psychological commitment is expressed in emotionally charged fan contexts. Secure attachment serves a protective, buffering role, whereas insecure attachment styles condition the expression of aggression by elevating baseline vulnerability and shaping responses to identity threat. These insights underscore the importance of considering individual differences in relational regulation when developing theoretical models and intervention strategies aimed at reducing aggression in sports fandom.

### Limitations

Several limitations of the present study should be acknowledged. First, the data were collected from a single city and included fans of teams that were randomly accessible within this context, which may limit the generalizability of the findings to other regions or cultural settings. Second, all variables were measured at a single time point, which precludes causal inferences and leaves the temporal ordering among psychological commitment, attachment styles, and aggression unresolved. Accordingly, the findings should be interpreted as reflecting associations and conditional relationships rather than directional or developmental effects. In addition, all measures were obtained via self-report questionnaires administered at the same time point, raising the possibility of social desirability bias and common method variance, which may have inflated the observed associations among variables. Although age, gender, and team allegiance were statistically controlled in the moderation analyses, other potentially relevant individual and contextual factors such as personality traits, impulsivity, emotion regulation strategies, alcohol use, and socioeconomic background were not included. These variables may jointly influence attachment orientations, psychological commitment, and aggressive tendencies, and their omission may limit the precision of the observed effects. Finally, although the moderation framework adopted in this study is theoretically grounded in attachment theory and prior research on emotion regulation and identity-related processes, the cross-sectional design restricts conclusions regarding temporal dynamics. Future research employing longitudinal, experimental, or cross-lagged designs is needed to examine how attachment orientations interact with psychological commitment over time and to clarify the conditions under which commitment is more or less likely to translate into aggressive behavior. Expanding future studies to include more diverse samples and additional psychological and situational covariates would further strengthen the external validity and theoretical refinement of this line of research.

### Implications

The findings of the present study offer several theoretical and practical implications for managing aggression in football fandom. First, the results suggest that interventions aimed at reducing fan aggression may benefit from incorporating psychological perspectives that account for individual differences in attachment-related emotion regulation, rather than focusing exclusively on situational or punitive approaches. In this respect, collaboration between the Ministry of Youth and Sports in Türkiye, mental health professionals, and sports organizations may facilitate the development of psychoeducational programs that enhance emotion regulation skills, self-awareness, and adaptive coping strategies among football supporters. Importantly, the present findings do not imply that attachment styles themselves should be directly targeted for change. Instead, secure attachment emerged as a protective regulatory orientation that buffers the expression of aggression among highly committed fans. Accordingly, intervention efforts may focus on strengthening functional capacities associated with attachment security such as emotional regulation, impulse control, empathy, and perspective-taking particularly in high-arousal and identity-threatening fan contexts. Group-based psychoeducation or counseling programs delivered to fans who have exhibited aggressive behavior may help reduce hostility by addressing these regulatory processes rather than attempting to alter underlying attachment orientations. At the institutional level, coordinated initiatives involving the Ministry of Youth and Sports, the Turkish Football Federation, and public health boards could support community-based projects that promote prosocial fan engagement and reduce intergroup hostility. Rather than targeting only sanctioned or penalized fans, inclusive social responsibility programs that bring together supporters from different teams may foster shared norms, reduce out-group demonization, and weaken the escalation of identity-based aggression. Such initiatives align with the present findings by addressing the social and emotional contexts in which aggression is most likely to emerge. For practitioners working in applied sport psychology, the results highlight the importance of considering fans’ psychological commitment and attachment-related regulatory tendencies when designing preventive strategies. Psychological counselors and sport psychologists may collaborate with athletes, clubs, and supporter groups to model adaptive responses to provocation, rivalry, and perceived injustice, thereby promoting healthier forms of identification and emotional expression within football culture. Finally, the study offers implications for future research. Scholars are encouraged to extend this line of inquiry by examining fan psychological commitment in relation to additional variables such as belongingness, trust, emotion regulation strategies, and coping styles, using longitudinal or experimental designs. Such approaches would allow for a more precise understanding of how individual differences interact with social and contextual factors to shape aggressive behavior in sports fandom.

## Data Availability

The raw data supporting the conclusions of this article will be made available by the authors, without undue reservation.
